# Transcriptome profiling of sulfate deprivation responses in two agarophytes *Gracilaria changii* and *Gracilaria salicornia* (Rhodophyta)

**DOI:** 10.1038/srep46563

**Published:** 2017-04-24

**Authors:** Wei-Kang Lee, Parameswari Namasivayam, Janna Ong Abdullah, Chai-Ling Ho

**Affiliations:** 1 Department of Cell and Molecular Biology, Faculty of Biotechnology and Biomolecular Sciences, Universiti Putra Malaysia, 43400 UPM-Serdang, Selangor, Malaysia

## Abstract

Seaweeds survive in marine waters with high sulfate concentration compared to those living at freshwater habitats. The cell wall polymer of *Gracilaria* spp. which supplies more than 50% of the world agar is heavily sulfated. Since sulfation reduces the agar quality, it is interesting to investigate the effects of sulfate deprivation on the sulfate contents of seaweed and agar, as well as the metabolic pathways of these seaweeds. In this study, two agarophytes *G. changii* and *G. salicornia* were treated under sulfate deprivation for 5 days. The sulfate contents in the seaweed/agar were generally lower in sulfate-deprivated samples compared to those in the controls, but the differences were only statistically significant for seaweed sample of *G. changii* and agar sample of *G. salicornia*. RNA sequencing (RNA-Seq) of sulfate-deprivated and untreated seaweed samples revealed 1,292 and 3,439 differentially expressed genes (DEGs; ≥1.5-fold) in sulfate-deprivated *G. changii* and *G. salicornia*, respectively, compared to their respective controls. Among the annotated DEGs were genes involved in putative agar biosynthesis, sulfur metabolism, metabolism of sulfur-containing amino acids, carbon metabolism and oxidative stress. These findings shed light on the sulfate deprivation responses in agarophytes and help to identify candidate genes involved in agar biosynthesis.

Inorganic sulfate is the most oxidized and dominant form of sulfur. It is an essential macronutrient for all organisms, which can be incorporated into amino acids, vitamins, cofactors, prosthetic groups, and a variety of primary and secondary metabolites^1^. Sulfur metabolism and the enzymes involved have been extensively studied in higher plants^2^. However, sulfur assimilation in algae remains largely unexplored but is presumably more similar to that in higher plants than in the prokaryotes, with a few exceptions on anion translocators^3^ and O-acetylserine (thiol)-lyase^4^. Briefly, inorganic sulfate transported into the cells is activated by ATP sulfurylase to form adenosine 5′-phosphosulfate (APS)^5^. Subsequently, APS can be either phosphorylated into 3′-phosphoadenosine 5′-phosphosulfate (PAPS) for the sulfation of metabolites, or converted into cysteine, methionine and glutathione for the synthesis of reduced sulfur compounds^6^.

Most photosynthetic organisms have limited sulfur storage in cells and depend solely on continuous uptake of sulfate from surrounding environment^7^. In freshwater and terrestrial environments, sulfate is often present at low concentration (10–50 μM) thus specific sulfate acclimation responses have been developed by different taxa. Transcriptomic responses towards sulfate deprivation had been extensively studied in photosynthetic organisms, such as *Arabidopsis thaliana*^8^,^9^,^10^,^11^, freshwater green alga *Chlamydomonas reinhardtii*^7^,^12^,^13^,^14^,^15^, cyanobacteria *Synechocystis sp.*^16^, and marine diatom *Emiliania huxleyi*^17^. The common responses during sulfate deprivation of these organisms include up-regulation of genes encoding sulfate transporters, increase in sulfate reducing capacity, synthesis of extracellular arylsulfatase and catabolism of sulfur-containing amino acids/secondary metabolites^8^.

In contrast with freshwater or terrestrial plants, seaweeds grow in marine waters with high sulfate concentrations ranging from 25 to 28 mM^17^ and hence sulfate may not be a limiting factor for cell growth and productivity in marine organisms. Many sulfated polysaccharides are synthesized by marine algae including agar^18^. Agar which is produced in the inner cell wall of red seaweeds may contribute to 6–71% of the total dry weight of seaweeds^19^. The backbone of agar is made up of two carbohydrate monomers D-galactose and L-galactose, with sulfate as the main side chain substitution^20^. Although the anionic matrix of sulfated polysaccharides provide heavily hydrated surface and higher flexibility enabling seaweeds to cope with dessication, strong ocean waves, osmotic stress, fluctuation in pH and temperature^18^, sulfation was found to lower agar gel strength^21^.

Seaweeds belonging to the genus *Gracilaria* contribute up to 60–80% of worldwide agar production^22^. Among these agarophytes, *G. changii* has a good agar yield (12–29% dry weight) and agar gel strength (180–563 g/cm^2^), while *G. salicornia* has a lower agar yield (9–14% dry weight) and poor gel strength (90–167 g/cm^2^)^23^,^24^,^25^,^26^. The molecular response of these red seaweeds to sulfate deprivation is largely unknown. We had previously shown that *G. changii* and *G. salicornia* collected during the rainy and dry seasons had minor increments in agar yields and gel strengths after sulfate deprivation for 5 days, compared to their respective controls, with a significant increase in agar yield for sulfate-deprivated *G. salicornia* collected during rainy season^25^. Although the effects of sulfate starvation on agar yield, gel strength, and other physical properties observed were not prominent, transcriptome sequencing of these samples may reveal dynamic changes in gene expression of these seaweeds to a sulfate free environment. In this study, we profiled and compared the transcriptomes of sulfate-deprivated *G. changii* and *G. salicornia* collected during rainy season whose yield and quality of agar were more affected by the sulfate deprivation treatment^25^. In addition, the transcriptomes of *G. changii* (with a higher agar yield and gel strength) and *G. salicornia* (with a lower agar yield and gel strength) were compared.

## Results and Discussion

### Effects of sulfate deprivation on the sulfate contents of seaweed and agar

**Figure 1:**
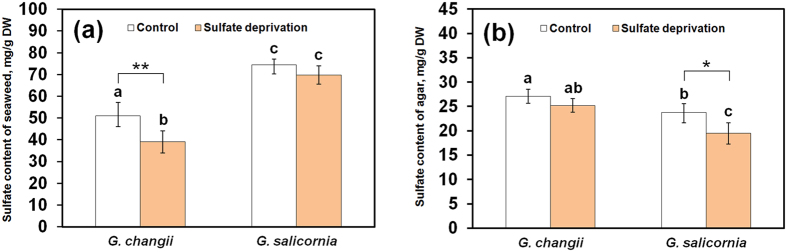
Sulfate content in the thalli of *G. changii* and *G. salicornia* (a) and their agars (b). DW, dry weight. Error bars denote ± standard deviation (SD), n = 5. Asterisks (*) and (**) show significant difference (*p* < 0.05) and highly significant difference (*p* < 0.01), respectively, between control and sulfate-deprivated samples. Different letters indicate significant differences (*p* < 0.05) analyzed by Duncan Multiple Range Test.

Marine environment has stable sulfate content since 40–50 million years ago^27^. The production of sulfated cell wall polysaccharides (such as agar) could be an adaptation strategy that many seaweeds acquired over geological time to avoid sulfate toxicity in an environment with high, unchanging sulfate. We were interested to find out whether the sulfation level of agar decreased when the sulfate content in seawater was removed. Our data (Fig. 1) showed that sulfate deprivation generally reduced the sulfate content in the two *Gracilaria* species and their agars, but the differences were only statistically significant in *G. changii* seaweed sample (*p* = 0.0095) and *G. salicornia* agar sample (*p* = 0.0243). The effects of species and treatment on the sulfate content in the seaweed/agar samples were statistically significant (*p* < 0.001; Supplementary Table S1). These findings suggested that the sulfate distribution into different biological pathways (including the sulfation of agar) may vary according to the sulfate content in the marine environment and in different *Gracilaria* species.

### *De novo* transcriptome assembly and functional annotation

RNA-Sequencing (RNA-Seq) produced a total of 51.8 to 53.7 million raw reads for *G. changii*. The total sequencing throughput for *G. salicornia* was in the range of 35.9–37.2 million raw reads. A summary of the transcriptome sequencing data and assembly is shown in Supplementary Table S2. After the quality filtering process, a high percentage of clean reads (>99%) was obtained for both species, indicating that the sequencing reads were of good quality. Velvet assembly of paired-end reads and iterative clustering of the contigs produced 15,846 and 20,671 unigenes for *G. changii* and *G. salicornia*, respectively (Table 1). Although the sequencing depth for *G. salicornia* samples was lower compared to that of *G. changii*, the total number of unigenes obtained from the RNA-Seq data of *G. salicornia* was about 30% higher.

The statistics on functional annotation of *G. changii* and *G. salicornia* unigenes are summarized in Table 1. A total of 10,341 unigenes (65.26%) from *G. changii* and 11,736 unigenes (56.78%) from *G. salicornia* have at least one match to the databases with an E-value < 10^−5^. However, the numbers of unigenes with Gene Ontology (GO) and Kyoto Encyclopedia of Genes and Genomes (KEGG) annotations are smaller compared to the numbers of unigenes that matched to the National Center for Biotechnology Information (NCBI) databases. *G. salicornia* has a higher number of unknown genes (43.22%) compared to *G. changii* (34.74%). The unannotated unigenes in these two *Gracilaria* species could be too short for similarity search or shared low similarities with annotated sequences in the databases.

**Figure 2:**
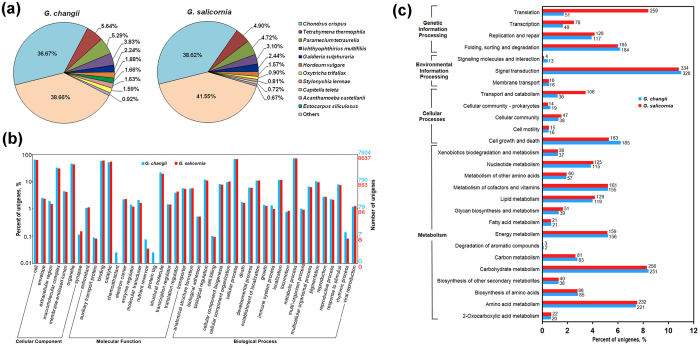
Functional annotation of unigenes from *G. changii* and *G. salicornia*. (**a**) Distribution of unigenes by species according to the best match of BLASTx results to non-redundant (NR) database at NCBI. (**b**) Gene Ontology (GO) annotation based on three main categories: cellular component, molecular function and biological process. (**c**) Kyoto Encyclopedia of Genes and Genomes (KEGG) pathways classification of the annotated unigenes.

Many annotated unigenes from *G. changii* (36.67%) and *G. salicornia* (38.62%) have high similarities to those in the red seaweed *Chondrus crispus* (Fig. 2a). However, some unigenes from these two *Gracilaria* species also share sequence similarity with the ciliated protozoa *Tetrahymena thermophila* (4.90–5.64%), *Paramecium tetraurella* (4.72–5.29%), *Ichthyophthirius multifiliis* (3.10–3.83%), *Stylonychia lemnae* (0.81–1.63%) and *Oxytrichia trifallax* (0.90–1.59%), which are taxonomically distant from red algal lineage. Since the transcriptomes were obtained from field samples, we do not exclude the possibility that some of these sequences may have originated from co-existing organisms/eukaryotes that were not removed by the cleaning process.

The distribution of GO categories for the two *Gracilaria* transcriptomes is very similar, with only minor differences observed (Fig. 2b). For example, unigenes assigned in two ontologies “chemoattractant activity” (GO: 0042056) and “protein tag” (GO: 0031386) were present in *G. changii* but absent in *G. salicornia*. Comparison of KEGG annotation between the two *Gracilaria* species showed that *G. changii* had a higher percentage of unigenes assigned to the category “cell growth and death” while *G. salicornia* had a higher percentage of unigenes assigned to “translation”, “transcription” and “transport and catabolism” categories (Fig. 2c). Reciprocal BLAST search of *G. changii* and *G. salicornia* unigenes revealed 4,018 homologous matches (Supplementary Table S3). We also identified a set of genes that are only expressed in *G. changii* (e.g., genes encoding galactose-2,6-sulfurylases I, polysaccharide pyruvyltransferase and thiosulfate sulfurtransferase rhodanese-like protein) or *G. salicornia* (e.g., genes for glutathione synthetase, glutamate dehydrogenase and carbon-sulfur lyase) (Supplementary Table S3). Some of these genes may be responsible for the major morphological and physiological differences (including the yield and quality of agar) between the two *Gracilaria* species.

### Differential gene expression analysis

In this study, RNA pooled from the thalli of three individual plants was sequenced. Although the differences in gene expression of individual seaweeds may not be captured, the gene expression and biological noise from individual biological replicates were averaged by pooling biological samples^28^. Biological averaging method, in which individual biological replicates were replaced with pooled biological replicates, has been employed in many real time PCR, microarray and RNA-Seq experiments^29^,^30^,^31^. On the other hand, gene expression data derived from high throughput sequencing are highly replicable^32^ and the pooled samples generally average the technical variation^33^.

About 89–95% and 87–95% of the short reads from *G. changii* and *G. salicornia*, respectively, were mapped to their respective unigenes, indicating that the assemblies were of good quality. In this study, only differentially expressed genes (DEGs) with a reads per kilobase per million reads (RPKM) value equal or above 15 were reported because more than 50% of the DEGs which was below this threshold do not have sequence similarity with red algae (Supplementary Fig. S1). This was performed to reduce the possibility of reporting false positive genes originating from other co-existing organisms. The vast majority of genes in this study do not have high expression fold-change in the samples being compared thus we used 1.5 expression fold-change as a threshold value in defining DEGs. Many interesting and relevant genes may not be included in the list of DEGs by setting a higher threshold value. Furthermore, the same threshold value has been used by other studies for the identification of DEGs^16^,^34^,^35^.

**Figure 3:**
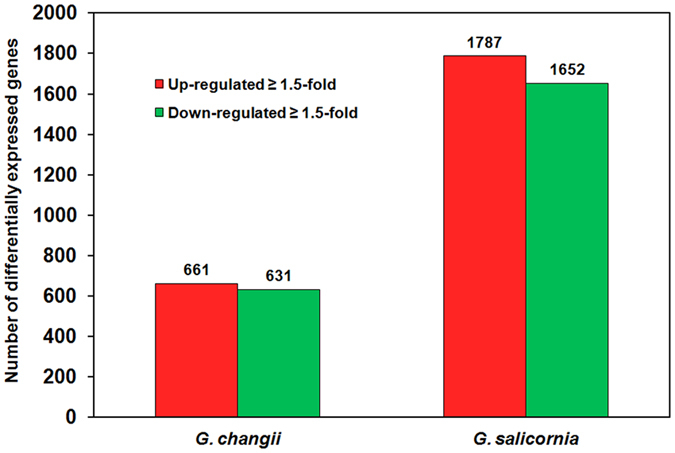
Number of up-regulated and down-regulated genes in *G. changii* and *G. salicornia* treated under sulfate deprivation for 5 days. Genes with a fold change of ≥1.5-fold in transcript abundance are considered as differentially expressed genes in sulfate-deprivated sample compared to the control sample.

In *G. changii*, 661 genes were up-regulated in sulfate-deprivated samples while 631 genes were down-regulated in sulfate-deprivated samples (Fig. 3). In *G. salicornia*, 1,787 genes were up-regulated in sulfate-deprivated samples while 1,652 were down-regulated in sulfate-deprivated samples (Fig. 3). The percentage of DEGs was higher in *G. salicornia* (16.64%) compared to that in *G. changii* (8.15%).

### Verification of DEGs with quantitative reverse transcriptase polymerase chain reaction (qRT-PCR)

**Figure 4:**
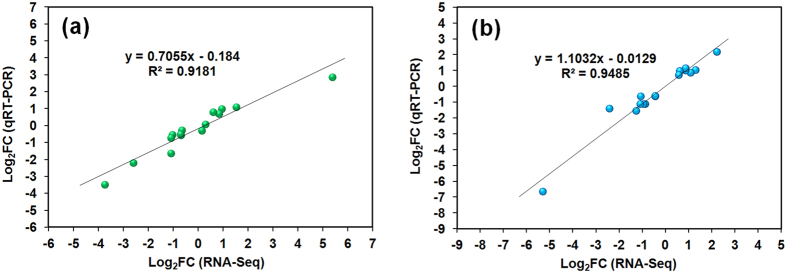
Verification of differentially expressed genes from *G. changii* (**a**) and *G. salicornia* (**b**) using qRT-PCR. A total of 15 DEGs from *G. changii* and *G. salicornia*, respectively, were verified. FC, fold change.

The expression of 15 genes from *G. changii* and *G. salicornia*, respectively, were verified by qRT-PCR. These randomly selected unigenes covered a wide range of RPKM values and log_2_Fold change (FC) values including two genes that were not differentially expressed. In general, the expression profiles of the unigenes in *G. changii* and *G. salicornia*, generated by RNA-Seq, corroborated with those obtained from qRT-PCR, with R^2^ values of 0.92 and 0.95 for *G. changii* and *G. salicornia*, respectively (Fig. 4). The results showed a high consistency between the results generated by RNA-Seq and qRT-PCR.

### Responses of *G. changii* and *G. salicornia* to sulfate deprivation

To understand the responses of red seaweeds to sulfate deprivation, the expression of DEGs involved in sulfur acquisition and assimilation, metabolism of sulfur-containing amino acids, transport systems, and putative agar biosynthesis (Tables 2 and 3) were analysed and discussed.

### Sulfate transport

Genes encoding ATP-binding cassette from *G. changii* (GC_457), S-type anion channel (GS_8662) and ABC transporters (GS_3784, GS_4151 and GS_660) from *G. salicornia* were found to be up-regulated in their sulfate-deprivated samples (Tables 2 and 3). It is unclear whether these ABC transporters participated in the transportation of exogenous sulfate into seaweeds. Two unigenes encoding putative sulfate permeases in the DASS/SLC13 permease family were identified in *G. changii* and *G. salicornia*, respectively, but were not being differentially expressed in response to sulfate deprivation. Meanwhile, the genes encoding high affinity H^+^/SO_4_^2−^ symporter (designed as SULTR in plants)^2^ were not identified in the transcriptomes of *G. changii* and *G. salicornia*. Our findings suggested that the sulfate transporters in *Gracilaria* species could be different from those reported in higher plants.

### Sulfur acquisition and assimilation

**Figure 5:**
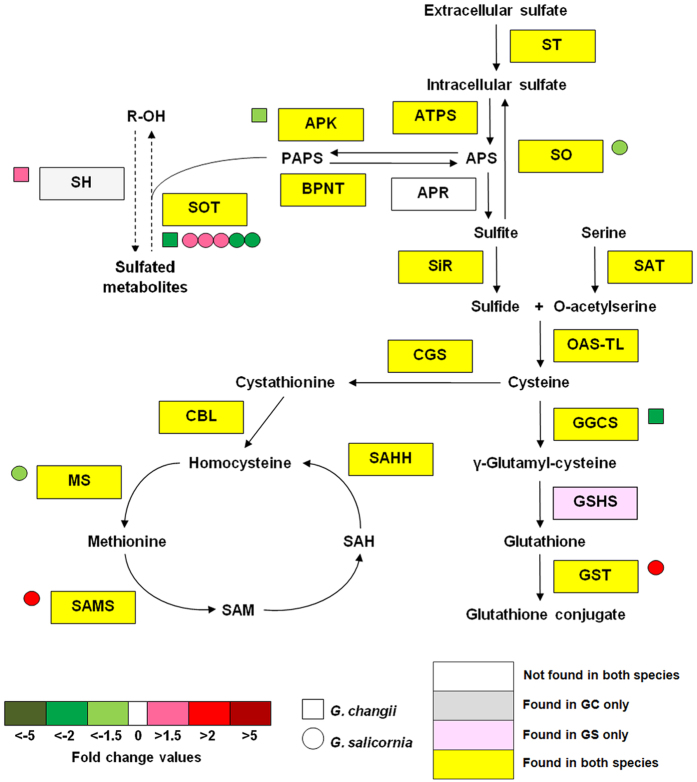
Effects of sulfate deprivation on the expression level of genes involved in sulfur metabolism. The sulfur metabolism pathway was modified from Takahashi *et al*.^2^. Each square and round box represents an unigene from *G. changii* (GC) and *G. salicornia* (GS), respectively. Yellow and white rectangles indicate transcripts that are found to be present or absent in the transcriptomes of both *Gracilaria* species, respectively, whereas grey and pink rectangles indicate transcripts that are only present in *G. changii* and *G. salicornia*, respectively. Dotted lines indicate enzymatic reaction encoded by unknown gene(s) from a gene family. All expression values are based on the biological averaging of pooled samples from three individual plants. APS, adenosine 5′-phosphosulfate; APK, APS kinase; APR, APS reductase; ATPS, ATP sulfurylase; BPNT, 3′(2′), 5′-bisphosphate nucleotidase; CBL, cystathionine beta-lyase; CGS, cystathionine gamma-lyase; GGCS, γ-glutamylcysteine synthetase; GSHS, glutathione synthetase; GST, gluthathione-S-transferase; MS, methionine synthase; OAST-TL, O-acetylserine (thiol)-lyase; PAPS, 3′-phosphoadenosine 5′-phosphosulfate; SAH, S-adenosylhomocysteine; SAHH, SAH hydrolase; SAM, S-adenosylmethionine; SAMS, SAM synthethase; SAT, serine acetyltransferase; SH, sulfohydrolase/sulfurylase; SiR, sulfite reductase; SO, sulfite oxidase; SOT, sulfotransferase; ST, sulfate transporter/permease.

Although the gene for ATP sulfurylase (ATPS) was identified in the *Gracilaria* species, it was not being differentially expressed in either species, suggesting that the transcription of this gene was not affected by sulfate-deprivation. APS is a branching point for sulfate assimilation into reductive or phosphorylative pathway^2^. Gene encoding the key enzyme in the reductive pathway, APS reductase was not found in the transcriptomes of both *Gracilaria* species (Fig. 5). It is unclear whether the seaweeds could obtain reduced sulfur through other enzymes/pathways, or the demand for the reduced sulfur was low during the experiments. The changes in transcript abundance for APS reductase in sulfate-deprivated *E. huxleyi*^17^ and *Ch. reinhardtii*^15^ were also non-significant compared to their respective controls. In a separate study^36^, the transcript encoding APS reductase from *Ch. reinhardtii* accumulated within 4 hours upon sulfur-deprivation before declining to a level comparable to that in the control, but its enzyme activity increased progressively over 24 hours, suggesting that this enzyme is regulated post-transcriptionally.

In this study, a gene encoding sulfite oxidase (GS_3043) was down-regulated in sulfate-deprivated *G. salicornia* (Table 3; Fig. 5). This gene was reported to be up-regulated in sulfate-deprivated *Ch. reinhardtii*^15^, regenerating free sulfate from excess sulfite recycled from cysteine and sulfur-containing amino acids. The down-regulation of this gene may suggest that regeneration of free sulfate from sulfite may reduce in sulfate-deprivated *G. salicornia* and sulfite is possibly an important intermediate for the biosynthesis of sulfide, cysteine and other sulfur-containing amino acids during sulfate deprivation.

Sulfate deprivation of *G. changii* decreased the transcript abundance of a gene encoding APS kinase (GC_2611) which is involved in sulfate phosphorylation (Table 2; Fig. 5). This suggested that sulfate deprivation may reduce PAPS production and subsequently the incorporation of sulfate into sulfur-containing metabolites (including agar), as shown by the slight decline in sulfate content in agar (Fig. 1b). However, APS kinase was not found to be differentially expressed in *G. salicornia* with significantly lower sulfate content in its agar (Fig. 1b) thus suggesting that there are other mechanism(s) that reduced the sulfate content in the agar of this species.

A decrease in the transcript abundance of APS kinase might lower the amount of cellular PAPS, which generally affects the transcriptional activity of sulfotransferases^37^. True enough, a gene encoding sulfotransferase 1C2A-like (GC_4742) from *G. changii* was down-regulated following sulfate deprivation (Table 2).

In *G. salicornia*, genes encoding heparan-sulfate-6-O-sulfotransferase (GS_1740) and carbohydrate sulfotransferase (GS_6282) were down-regulated in sulfate-deprivated sample (Table 3; Fig. 5). The decrease of these two transcripts in sulfate-deprivated *G. salicornia* coincided with a significant decrease of sulfate content in its agar (Fig. 1b). However, the involvement of these two carbohydrate sulfotransferases in the sulfation of galactose sugar requires further verification. On the other hand, the expressions of three sequences encoding glycolipid sulfotransferases (GS_8537, GS_8540 and GS_8541) were up-regulated in sulfate-deprivated *G. salicornia* suggesting that the demand for sulfation in glycolipid could be higher than that in the carbohydrate, when the seaweeds were deprived of sulfate sources.

### Redistribution and recycling of sulfur

Extracellular arylsulfatase was found to be up-regulated in the green alga *Ch. reinhardtii*^15^,^38^ and marine diatom *E. huxleyi*^17^ to recycle sulfate from alternative sources during sulfate deprivation. In this study, gene encoding arylsulfatase was not found in the transcriptomes of *Gracilaria* species. However, the transcript abundance of a gene encoding galactose-2,6-sulfurylase I (GC_13944) was increased by 1.86 fold in sulfate-deprivated *G. changii* (Table 2; Fig. 5). The changes in gene expression coincide with the minor reduction of sulfate content in the agar samples of sulfate-deprivated *G. changii*, suggesting the putative role of galactose-2,6-sulfurylase I in recycling sulfur from sulfated galactans. Galactose-2,6-sulfurylase is a novel enzyme which has only been reported in the red alga *C. crispus*, and was proposed to catalyse the conversion of D-galactose-6-sulfate into 3,6-anhydro-D-galactose^39^. However, the transcripts encoding galactose-2,6-sulfurylase I were not found in the transcriptome of *G. salicornia*. It is unknown whether a higher gel strength in *G. changii* agar compared to that in the *G. salicornia* was attributed to the gene expression and activity of the species-specific galactose-2,6-sulfurylase I. Genes encoding rhodanese and cysteine/taurine dioxygenase had been reported to be involved in the recycling of internal sulfur^15^, but they were not differentially expressed in both *Gracilaria* species.

### Metabolism of glutathione and methionine

Overall, the genes involved in the metabolism of glutathione and methionine showed different responses in sulfate-deprivated *G. changii* and *G. salicornia*. The gene encoding γ-glutamylcysteine synthetase (GC_2731), which is responsible for the biosynthesis of L-γ-glutamylcysteine from L-cysteine and L-glutamate, was down-regulated in sulfate-deprivated *G. changii* (Table 2; Fig. 5). Meanwhile, the enzyme which catalyses the biosynthesis of glutathione from L-γ-glutamylcysteine was not found in the transcriptome of *G. changii* (Fig. 5). Our results suggested that the glutathione synthesis in *G. changii* could be depressed by sulfate deprivation in *G. changii.* The genes encoding for γ-glutamylcysteine and glutathionine synthetase were not differentially expressed in sulfate-deprivated *G. salicornia* (Fig. 5).

A gene encoding peptide methionine sulfoxide reductase (GC_2663) was down-regulated in sulfate-deprivated *G. changii* (Table 2). This result was in contrast with that in *G. salicornia*, in which the unigene encoding peptide methionine sulfoxide reductase (GS_3594) was up-regulated in response to sulfate deprivation (Table 3). The increase in the abundance of transcript encoding peptide methionine sulfoxide reductase (which catalyses the regeneration of methionine from methionine sulfoxide) and methionine aminopeptidase (GS_14648) in sulfate-deprivated *G. salicornia* suggest that methionine might be recycled from proteins during sulfate deprivation.

Meanwhile, a gene encoding methionine synthase (GS_4274) was down-regulated in sulfate-deprivated *G. salicornia* while the transcript encoding S-adenosylmethionine (SAM) synthethase (GS_4407) was accumulated in its sulfate-deprivated samples (Table 3). This suggested that under sulfate deprivation, methionine was recycled from proteins and converted to SAM which is important for methylation process. The response was similar to that of *A. thaliana* treated under sulfate deprivation^9^, but is in contrast with those in sulfate-deprivated *G. changii, Ch. reinhardtii*^15^ and *E. huxleyi*^17^.

### Biosynthesis of dimethylsulfoniopropionate (DMSP)

DMSP is an osmolyte and cryoprotectant accumulated in many algal species in response to abiotic stress and nutrient deprivation^40^,^41^,^42^. DMSP biosynthesis is dependent on APS reductase activity which regulates the synthesis of its precursor, methionine, through the reductive pathway of sulfur assimilation^43^. Genes involved in the biosynthesis of DMSP have been identified and characterized, including 2-oxoglutarate-dependent aminotransferase, NADPH-linked reductase and SAM-dependent methyltransferase^44^. These genes were not being found (2-oxoglutarate-dependent aminotransferase and NADPH-linked reductase) or being differentially expressed (SAM-dependent methyltransferase) in *G. changii* and *G. salicornia* possibly due to a relatively low content of DMSP in *Gracilaria* spp. compared to that in green algae and phytoplankton^45^,^46^.

### Putative agar biosynthesis

**Figure 6:**
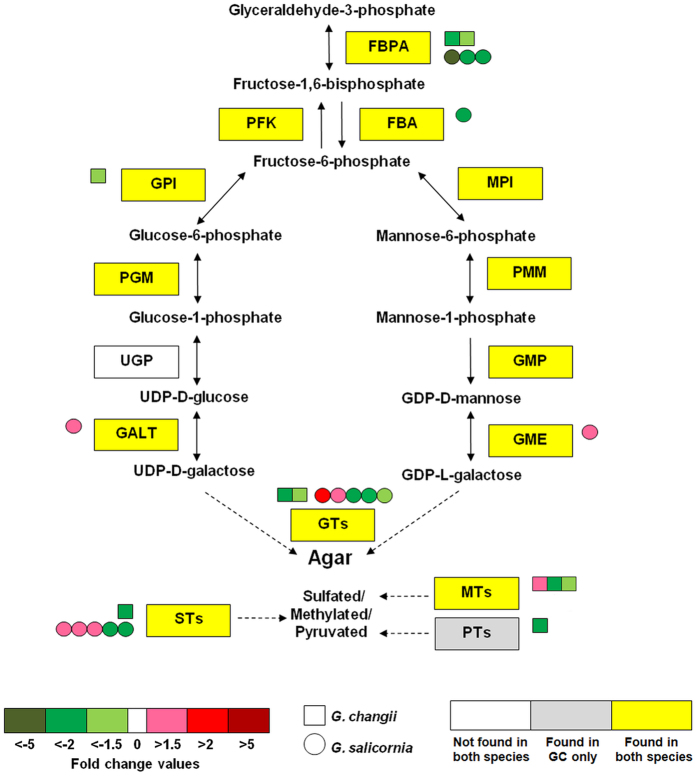
Effects of sulfate deprivation on the expression level of putative genes involved in agar biosynthesis. The putative agar biosynthetic pathway was modified from Lee *et al*.^47^. Each square and round box represents an unigene from *G. changii* (GC) and *G. salicornia* (GS), respectively. Yellow and white rectangles indicate transcripts that are found to be present or absent in the transcriptomes of both *Gracilaria* species, respectively, whereas grey rectangle indicates transcripts that are present in *G. changii* only. Dotted lines indicate enzymatic reaction encoded by unknown gene(s) from a gene family. All expression values are based on the biological averaging of pooled samples from three individual plants. FBPA, fructose-1,6-bisphosphate aldolase; FBA, fructose-1,6- bisphosphatase; GALT, galactose-1-phosphate uridylyltransferase; GME, GDP-mannose-3′,5′-epimerase; GMP, GDP-mannose pyrophosphorylase; GPI; glucose-6-phosphate isomerase; GTs, glycosyltransferases; MPI; mannose-6-phosphate isomerase; MTs, methyltransferases; PFK, phosphofructokinase; PGM, phosphoglucomutase; PMM, phosphomannose mutase; PTs, pyruvyltransferases; STs, sulfotransferases; UGP, UTP-glucose-1-phosphate uridylyltransferase.

The biosynthetic pathway of agar is less explored and the genes involved have yet been elucidated^47^. In this study, we identified a number of putative genes which could be involved in the biosynthesis of agar precursors from *G. changii* and *G. salicornia* (Fig. 6). DEGs related to the biosynthesis of fructose-6-phosphate (a central metabolite used for the synthesis of agar monomer), elongation of polysaccharide chain and modification of its side chain substitutions were found in sulfate-deprivated samples (Fig. 6). The agar yield of sulfate-deprivated *G. salicornia* increased significantly^25^, coincides with the increase in the transcript abundance of genes encoding galactose-1-phosphate uridylyltransferase and GDP-mannose-3′,5′-epimerase (Table 3; Fig. 6), suggesting their putative roles in regulating the biosynthesis of agar precursors. The gene expression level of these two genes had been shown to correlate with the agar content in *Gracilaria* and *Gracilariopsis* samples^24^,^48^,^49^.

In addition, most of the genes encoding galactosyltransferases (GTs), sulfotransferases (STs), methyltransferases (MTs) and pyruvyltransferase (PTs) were down-regulated in *G. changii* in response to sulfate deprivation (Fig. 6). In *G. salicornia*, some of the genes encoding GTs and STs showed different expression patterns from those in *G. changii*, they were being up-regulated in response to sulfate deprivation (Fig. 6). However, the gene encoding PT was not being identified in sulfate-deprivated *G. salicornia*. The differential expression of these genes in *G. changii* and *G. salicornia* may lead to higher gel strength in the former, as sulfation and methylation on the agar were reported to produce a weaker gel^50^,^51^. In most organisms, glycosyltransferases (including glucosyltransferases and galactosyltransferases) represent a large diverse group of enzymes with more than 30 gene families^52^. To our knowledge, glycosyltransferase which is responsible for agar polymerization has not been identified. In this study, we have identified a few DEGs encoding glycosyltransferase. Two of them (GS_4319 and GS_6663) were up-regulated in sulfate-deprivated *G. salicornia* sample (Table 3 and Fig. 6), which had an increase in agar yield upon sulfate deprivation^25^.

### Other pathways

The genes involved in photosynthesis, such as those encoding phycobilisome linker polypeptide, photosystem II Q and light harvesting protein, were differentially expressed in sulfate-deprivated *G. changii* and *G. salicornia*, with different expression patterns (Tables 2 and 3). In *Ch. reinhardtii*, the genes encoding different light harvesting proteins have different expression patterns under sulfate deprivation^15^, suggesting that these proteins may respond differently to sulfate deprivation.

Sulfate deprivation regulated a number of genes involved in carbon metabolism, suggesting a tight interconnection between carbon and sulfur metabolisms. For example, genes encoding transaldolase, fructose-1,6-bisphosphate aldolase, and glycoside hydrolases were differentially expressed in sulfate-deprivated *G. changii* and *G. salicornia*, but the expression patterns were species-dependent (some of these genes were up-regulated in one species but down-regulated in another species, and vice versa) (Tables 2 and 3). There are a number of genes that were only differentially expressed in sulfate-deprivated *G. changii* or *G. salicornia*, such as genes encoding glucose-6-phosphate isomerase (GC_1234), sedopheptulose-1,7-bisphosphatase (GC_7206), glyceraldehyde-3-phosphate dehydrogenase (GS_5744 and GS_6394), phosphoglycerate kinase (GS_5992) and 6-phosphogluconate dehydrogenase (GS_1698) (Tables 2 and 3).

The genes encoding superoxide dismutase (GC_2437) and glutathione S-transferase (GS_5546) from *G. changii* and *G. salicornia*, respectively; were up-regulated in sulfate-deprivated sample (Tables 2 and 3), similar to that observed in sulfate-deprivated *A. thaliana*^9^. Meanwhile, a few transcripts encoding haloperoxidase-like proteins, including vanadium-dependent bromoperoxidases, were decreased by sulfate deprivation in both *Gracilaria* species. Although the biological role of haloperoxidase-like protein remains unclear, these genes have been shown to be differentially expressed in red seaweeds under various abiotic stresses, such as light, salinity and temperature^53^,^54^,^55^. It is noteworthy that the increase of these stress-related transcripts could be a general stress response.

## Conclusions

The findings of this study offer a snapshot of the global transcriptional responses upon sulfate deprivation in two *Gracilaria* species, i.e. *G. changii* and *G. salicornia*. Among the DEGs were genes involved in putative agar biosynthesis, sulfur acquisition and assimilation, metabolism of sulfur-containing amino acids, transport systems and oxidative stress. These two species responded differently to sulfate deprivation, possibly due to differences in their cellular sulfate storage and demand as well as synthesis of sulfated polysaccharides, thus affecting the expression of genes encoding enzymes related to sulfate phosphorylation, removal of sulfate, methionine and SAM cycle, and recycling of sulfur. In addition, sulfate deprivation responses of *Gracilaria* species were found to be different from those in *Ch. reinhardtii, E. huxylei* and *A. thaliana*, suggesting a unique acclimation response in marine macroalgae. However, these differences could also be contributed by gene expression profiling methods used, physiological states of the sample, experimental design, severity of sulfate deprivation, and duration of treatment.

## Materials and Methods

### Seaweed materials and sulfate deprivation treatments

Collection of seaweed materials and sulfate deprivation treatment were performed as described by Lee *et al*.^25^. Briefly, *Gracilaria changii* (Xia *et* Abbott) Abbott, Zhang *et* Xia and *G. salicornia* (C. Agardh) Dawson without any cystocarpic structures were collected from the mangrove swamp at Morib, Selangor, Malaysia (02°45.878′ N; 101°25.976′ E) in October 2012 (rainy season). The seaweeds were washed from visible mud, epiphytes and epibionts, before acclimating in aerated aquaria containing 30 part per thousand (ppt) artificial seawater (ASW: Instant Ocean Synthetic Sea Salt, Aquarium Systems Inc., Mentor, OH, USA) at pH 7 and 25 °C.

Sulfate deprivation treatment was conducted on *G. changii* (CC, control sample; CT, treated sample) and *G. salicornia* (SC, control sample; ST, treated sample). The thalli of *G. changii* and *G. salicornia* were treated for 5 days in aerated aquaria containing sulfate-free ASW (450 mM NaCl, 370 mM KCl, 9 mM CaCl_2_.2H_2_O, 49 mM MgCl_2_.6H_2_O and 2 mM NaHCO_3_), with a control in normal ASW (450 mM NaCl, 370 mM KCl, 9 mM CaCl_2_.2H_2_O, 23 mM MgCl_2_.6H_2_O, 26 mM MgSO_4_.7H_2_O and 2 mM NaHCO_3_). The seaweeds were treated for five days because we could not maintain the seaweeds in a healthy condition (without necrosis) in the laboratory for a longer period. Growing in a marine environment with high and stable sulfate content, the seaweeds may have accumulated sufficient sulfate that may last for sometime. Due to that, we have chosen a sulfate-free treatment for a short duration (5 days). The treatment was performed under laboratory condition with natural photoperiod (12:12 h light:dark cycle, 8–10 μmol photons m^−2^s^−1^ during the light cycle), while the temperature (25 °C), pH (7.8) and salinity of seawater (35 ppt) were kept constant throughout the experiment. All the samples were collected from the field or harvested during day time (1200–1400 local time) to minimize the diurnal patterns among the samples.

### Measurement of sulfate content

The total sulfate content in seaweed and agar (that were pooled from thalli of a few individual plants, respectively) was determined using a modified BaCl_2_-gelatin turbidimetric method^56^. Approximately 15 and 40 mg of dried seaweed and agar samples, respectively, were hydrolysed in 0.5 M HCl (in a ratio of 1:150 w/v) at 110 °C for 10 hours. The solution was cooled to room temperature and filtered through Whatman No. 1 filter paper (Whatman, Hillsboro, OR). The seaweed or agar hydrolysate (0.2 ml each) was added to 3.8 ml of 3% (w/v) trichloroacetic acid and 1 ml of BaCl_2_-gelatin reagent (40 mM BaCl_2_, 0.3% w/v gelatin), mixed vigorously and incubated at room temperature for 30 min. The sample was replaced with 0.5 M HCl in the blank, while a “control” which consisted of gelatin solution without BaCl_2_ was prepared for each sample to eliminate artifacts contributed by ultraviolet light-absorbing materials produced during acid hydrolysis. Spectrophotometric measurement for each sample and its “control” was performed against the blank solutions at an absorbance of 360 nm. The optical reading obtained from the “control” was subtracted from that of the seaweed or agar hydrolysate before the sulfate content was calculated from a standard curve prepared with 1.15–5.74 mM K_2_SO_4_, per mg of seaweed or agar.

### Statistical analyses

All statistical analyses were performed using SAS statistical software version 9.3 (SAS Institute Inc., Cary, North Carolina, USA). Two-way ANOVA test was used to determine the significant effects of species and treatment, and their interactions on the sulfate content in seaweed and agar. Statistical difference in sulfate content between the control and sulfate-deprivated samples (from five replicates, respectively) was determined with Student’s *t*-test while Duncan Multiple Range Test (DMRT) was used to determine the significant differences among the experimental groups (e.g. CC, CT, SC and ST).

### Isolation and quality assessment of RNA

Total RNA was isolated from 10 g of each *Gracilaria* sample (i.e., CC, CT, SC and ST) which was pooled from the thalli of three individual plants each, as described by Chan *et al*.^57^. Genomic DNA was removed using RNase-free DNase I (New England Biolabs, Beverly, MA) following the manufacturer’s instructions. The purity and quality of the RNA were evaluated using Nanodrop spectrophotometer (Implen UK Ltd, Essex, UK) and Agilent 2100 Bioanalyzer (Agilent Technologies, USA). The RNA samples which met the following criteria were processed for RNA-Seq and qRT-PCR analyses: absorbance ratios A_260/280_ and A_260/230_ that were between 1.9–2.1 and more than 2, respectively, 25 S rRNA:18 S rRNA ratio near to 2:1 and RNA Integrity Number (RIN) more than 6.5.

### RNA-Seq, *de novo* assembly and annotation of the transcriptomes

Massive paired-end RNA Seq was performed on each *Gracilaria* sample using Illumina HiSeq 2000 platform (Illumina, San Diego, CA, USA) at a read length of 90 bp. The RNA-Seq data were deposited in European Nucleotide Archive (ENA) under the accession number PRJEB13899. The raw reads obtained from RNA-Seq were analysed with FastX toolkit v0.0.13.2 (http://hannonlab.cshl.edu/fastx_toolkit/) to remove low-quality reads with Phred quality scores <20, adaptor and ambiguous bases ‘N’. *De novo* assembly of clean reads obtained from the four samples was performed with Velvet (v1.2.08)^58^ with default parameters and optimized *k*-mers at 67, 69, 65, and 65 for CC, CT, SC, and ST, respectively. Assembled sequences that were less than 100 nucleotides were discarded. Iterative clustering of contigs from control and treated samples was performed with The Gene Index Clustering Tool (TGICL)^59^ to generate two sets of non-redundant contigs (unigenes) for *G. changii* and *G. salicornia*, respectively.

Sequence based alignments were performed against the following public databases: non-redundant protein sequences (NR), UniProtKB/SwissProt (Swiss-Prot), GO and KEGG. BLASTx program^60^ with default settings and a cut-off E-value of 10^−5^ was used to match the nucleotide sequences to those in the NR and SwissProt databases at the NCBI. The GO annotation was performed with BLAST2GO^61^ based on the most significant SwissProt matches and the results were visualized using Web Gene Ontology Annotation Plot (WEGO)^62^. KEGG annotation was performed with the KEGG Automatic Annotation Server (KAAS, http://www.genome.jp/kegg/kaas/)^63^. The most significant and informative matches were used to assign putative function to each unigene. Reciprocal BLASTn^64^ was conducted on the two sets of unigene with a cut-off E-value of 10^−30^.

### Identification of differentially expressed genes (DEGs)

The paired-end RNA-Seq reads from each sample were mapped to respective transcriptomes with Bowtie2^65^. The number of reads mapped to each unigene was converted into RPKM^66^, to normalise the differences in transcript length and sequencing depth. DEGs between control and sulfate-deprivated samples of *G. changii* and *G. salicornia*, respectively, were identified by DEGseq^67^, an R package which uses the MA-plot-based method with Random Sampling model (MARS). In the absence of replicates, DEGseq treated the counts of reads that were mapped to a gene from two samples (control and sulfate-deprivated samples in this study) as independent values and tested them with the following hypotheses, H0: p1 = p2 = p versus H1: p1≠p2; p1 and p2 denote the probability that a read obtained from sample 1 (e.g. control) and 2 (e.g. sulfate-deprivated), respectively. The two estimates generated from above were then used for the calculation of Z-score which can be converted to two-sided *p*-value^67^. Transcripts with RPKM read ≥15 in at least one of the samples, *p*-value less than 0.001 and fold change ≥1.5-fold were defined as DEGs (Supplementary Table S4).

### Verification of DEGs using qRT-PCR

Verification of DEGs was performed on the same RNA samples for RNA-Seq experiment. Affinity Script QPCR cDNA Synthesis Kit (Agilent Technologies, USA) was used to reverse transcribe 2.5 μg of total RNA from each sample into the first strand cDNA. The DEGs and the primers used for qRT-PCR analysis are listed in Supplementary Table S5. Primer3 software ver. 0.4.0 (http://frodo.wi.mit.edu)^68^ was used to design primers that are specific to the 3′-untranslated region flanking the open reading frame, to avoid amplification from possible isoforms/gene family members. Melting curve and standard curve (with serial dilutions of cDNA samples) were generated to check for amplification of single PCR product, absence of primer dimers and PCR efficiency (90–105%).

All qRT-PCR analyses were performed using Brilliant III Ultra-Fast SYBR Green QPCR Master Mix (Agilent Technologies, USA) and iQ^TM^5 real time detection system (Bio-Rad, USA). The reaction mixture (20 μl) consisted of 100 ng of first-strand cDNA template, 1X Brilliant III Ultra-Fast SYBR Green QPCR master mix, 200–400 nM of each respective primers and molecular grade H_2_O (bioWORLD, USA). The PCR amplification was performed with the following conditions: initial denaturation at 95 °C for 3 min, followed by 40 cycles of 95 °C for 5 sec and 60 °C for 10 sec; and a melt curve analysis with temperature increasing from 55 °C to 95 °C with an increment of 0.5 °C/10 sec. A control sample without template was included in all experiments to rule out the presence of contaminants and primer-dimer.

Two internal reference genes for *G. changii* (encoding histone and cytosolic fructose-1,6-biphosphatase, respectively) and *G. salicornia* (encoding 40S ribosomal protein S15a and tRNA-dihydrouridine synthase-1-like, respectively) were selected based on their stability ranking and pairwise variation as analysed by geNorm version 3.5^69^. All samples in the qRT-PCR analysis were performed in three technical replicates. The raw Ct values obtained were analysed using 2^−ΔΔCt^ method^70^,^71^, and normalized against the expression of internal reference genes. The normalized expression level in all samples was expressed as relative fold change to that in the control samples.

## Additional Information

**How to cite this article**: Lee, W.-K. *et al*. Transcriptome profiling of sulfate deprivation responses in two agarophytes *Gracilaria changii* and *Gracilaria salicornia* (Rhodophyta). *Sci. Rep.* **7**, 46563; doi: 10.1038/srep46563 (2017).

**Publisher's note:** Springer Nature remains neutral with regard to jurisdictional claims in published maps and institutional affiliations.

## Supplementary Material

Supplementary Information

Supplementary Table S3

Supplementary Table S4

## Figures and Tables

**Table 1:** Statistics of clustering and functional annotation of unigenes.

	*G. changii*	*G. salicornia*
**Clustering of unigenes**		
Total no. of unigenes	15,846	20,671
Total length (bp)	21,651,714	24,587,285
GC (%)	50.20	51.35
N50	3,295	2,931
**Annotation of unigenes**		
No. of annotated unigenes	10,341 (65.26%)	11,736 (56.78%)
No. of NCBI NR annotation	10,329 (65.18%)	11,724 (56.72%)
No. of NCBI Swiss-Prot annotation	8,144 (51.39%)	9,013 (43.60%)
No. of GO annotation	7,904 (49.88%)	8,637 (41.78%)
No. of KEGG annotation	2,977 (18.78%)	3,083 (14.91%)

**Table 2:** Expression level of selected differentially expressed genes from *G. changii.*

Functional category	Unigene ID	Putative function	Fold change expression (−S/+S)	*p* value
Sulfur metabolism	GC_45	S-adenosylmethionine carrier	1.95	0
	GC_13944	Galactose-2,6-sulfurylases I/L-amino-acid oxidase	1.87	1.19E-83
	GC_4742	Sulfotransferase 1C2A-like	−2.89	1.15E-28
	GC_2731	Gamma-glutamylcysteine synthetase	−2.11	0
	GC_2611	Adenosine-5 -phosphosulfate kinase	−1.60	4.50E-61
	GC_2663	Peptide methionine sulfoxide reductase	−1.57	7.34E-59
Carbon metabolism	GC_31	Capsular polysaccharide biosynthesis protein	1.80	0
	GC_3210	Glycoside hydrolase family GH16	−3.43	4.06E-257
	GC_5787	Glycoside hydrolase family GH16	−3.07	8.73E-299
	GC_4229	Glycoside hydrolase	−1.56	2.02E-105
	GC_7206	Sedoheptulose-1,7-bisphosphatase	−2.03	3.50E-26
	GC_2812	Fructose-bisphosphate aldolase	−2.07	7.14E-11
	GC_2356	Fructose-1,6-biphosphate aldolase	−1.65	1.22E-13
	GC_1954	Transketolase	−1.77	0
	GC_447	Transaldolase	−1.57	0
	GC_1234	Glucose-6-phosphate isomerase	−1.69	1.04E-104
	GC_12148	Nucleotide sugar transporter	−1.53	2.04E-38
Photosynthesis	GC_2467	Photosystem II protein	−1.83	5.74E-49
	GC_1245	Light-harvesting protein	−1.59	2.69E-31
Transferase activity	GC_3697	Methyltransferase-like protein	1.53	1.01E-11
	GC_2419	Beta-1,4-mannosyltransferase	−2.75	6.79E-20
	GC_2746	Glycosyltransferase/Methyltransferase family	−2.46	8.44E-162
	GC_3116	Polysaccharide pyruvyltransferase	−2.04	0
	GC_7710	Possible glycosyltransferase	−1.66	5.99E-33
	GC_4706	Methyltransferase	−1.64	6.71E-42
Peroxidase activity	GC_3015	PAP2/haloperoxidase-like protein	−3.71	0
	GC_5858	PAP2/haloperoxidase-like protein	−2.93	0
	GC_5283	Vanadium-dependent bromoperoxidase	−2.69	0
Others	GC_457	ATP-binding cassette, sub-family D (ALD), member 3	1.69	0
	GC_2437	Superoxide dismutase	1.52	1.09E-153
	GC_2930	Alkaline phosphatase	−1.72	2.52E-154

Genes with a fold change ≥1.5-fold in transcript abundance are considered as up-regulated/down-regulated genes in sulfate-deprivated sample (−S) compared to the control sample (+S). The up- or down-regulated genes are classified into different functional categories. All expression values are based on the biological averaging of pooled samples from three individual plants.

**Table 3:** Expression level of selected differentially expressed genes from *G. salicornia.*

Functional category	Unigene ID	Putative function	Fold change expression (−S/+S)	*p* value
Sulfur metabolism	GS_5546	Glutathione S-transferase	2.45	0
	GS_4407	S-adenosyl-methionine synthetase	2.13	0
	GS_3594	Peptide methionine sulfoxide reductase	1.75	2.32E-15
	GS_8540	Glycolipid sulfotransferase	1.82	1.5E-299
	GS_8537	Glycolipid sulfotransferase	1.75	6.48E-61
	GS_8541	Glycolipid sulfotransferase	1.66	1.26E-16
	GS_14648	Methionine aminopeptidase	3.07	1.2E-286
	GS_463	Methionine aminopeptidase	−1.95	1.1E-144
	GS_1740	Heparan-sulfate-6-O-sulfotransferase	−2.73	5.7E-119
	GS_6282	Carbohydrate sulfotransferase	−2.14	9.32E-48
	GS_4274	Methionine synthase	−1.66	5.1E-102
	GS_3043	Sulfite oxidase	−1.57	7.44E-42
Carbon metabolism	GS_3189	Cellulose synthase (UDP-forming)	6.06	0
	GS_5744	Glyceraldehyde-3-phosphate dehydrogenase	2.22	0
	GS_2763	Isoamylase glycoside hydrolase/glycogen debranching protein	2.14	8E-148
	GS_7955	Transaldolase	2.13	0
	GS_5748	Capsular polysaccharide biosynthesis protein	1.82	1.88E-44
	GS_1698	6-phosphogluconate dehydrogenase	1.58	0
	GS_5992	Phosphoglycerate kinase	1.51	4.48E-94
	GS_8637	GDP-mannose-3′,5′-epimerase	1.54	1.1E-166
	GS_3763	Galactose-1-phosphate uridylyltransferase	1.50	1.8E-139
	GS_2523	Fructose-1,6-biphosphate aldolase	−5.39	1.1E-142
	GS_5566	Fructose bisphosphate aldolase	−2.25	3.88E-07
	GS_2287	Fructose-1,6-biphosphate aldolase	−2.03	2.2E-150
	GS_198	Fructose-1,6-bisphosphatase precursor	−2.16	2.7E-112
	GS_6394	Glyceraldehyde-3-phosphate dehydrogenase	−3.51	3E-231
	GS_5942	Phosphoglycerate kinase	−2.62	6.33E-92
	GS_2163	Glycoside hydrolase	−2.07	1.5E-172
	GS_5649	Ribulose- bisphosphate carboxylase oxygenase large subunit N-methyltransferase	−1.53	1.65E-20
Photosynthesis	GS_2365	Phycobilisome 31.8 kD linker polypeptide	2.14	0
	GS_348	Photosystem II Q	1.84	3.75E-20
Transferase activity	GS_6663	Glycosyltransferase	3.53	8.33E-24
	GS_4319	Glycosyltransferase	1.66	1.3E-213
	GS_4258	Glycosyltransferase/methyltransferase family	−3.51	4.3E-218
	GS_152	Glycosyltransferase-like protein	−2.00	2.54E-43
	GS_1777	Glycosyltransferase	−1.87	2.5E-227
Peroxidase activity	GS_2825	PAP2/haloperoxidase-like protein	4.03	0
	GS_9077	Vanadium-dependent bromoperoxidase	−2.41	0
	GS_3842	PAP2/haloperoxidase-like protein	−2.93	7.1E-50
	GS_1594	PAP2/haloperoxidase-like protein	−2.20	3.76E-15
Others	GS_8662	S-type anion channel	2.19	0
	GS_4151	ABC transporter, ATP-binding protein	1.91	3.19E-83
	GS_6461	MYB domain-containing protein	1.83	2.6E-116
	GS_1272	MFS transporter, anion:cation symporter	1.71	9.67E-27
	GS_660	ABC transporter related protein	−1.87	4.8E-188
	GS_5959	Mitochondrial carrier protein/S-adenosylmethionine carrier	−1.87	1.4E-102
	GS_3784	ABC transporter permease	−1.84	7.4E-120

Genes with a fold change ≥1.5-fold in transcript abundance are considered as up-regulated/down-regulated genes in sulfate-deprivated sample (−S) compared to the control sample (+S). The up- or down-regulated genes are classified into different functional categories. All expression values are based on the biological averaging of pooled samples from three individual plants.
