# Supplementary material for: Transcriptome profiling of sulfate deprivation responses in two agarophytes Gracilaria changii and Gracilaria salicornia (Rhodophyta)
Source: Sci Rep. 2017 Apr 24;7:46563. doi: 10.1038/srep46563 (PMC5402284; doi:10.1038/srep46563)
Supplement: Supplementary Information [file srep46563-s1.pdf]

Transcriptome profiling of sulfate deprivation responses in two agarophytes *Gracilaria changii* and *Gracilaria salicornia* (Rhodophyta)

Wei-Kang Lee, Parameswari Namasivayam, Janna Ong Abdullah, Chai-Ling Ho*

Department of Cell and Molecular Biology, Faculty of Biotechnology and Biomolecular Sciences, Universiti Putra Malaysia, 43400 UPM-Serdang, Selangor, Malaysia.

*Corresponding author

Professor Dr. Chai-Ling Ho

Department of Cell and Molecular Biology, Faculty of Biotechnology and Biomolecular Sciences, Universiti Putra Malaysia, 43400 UPM-Serdang, Selangor, Malaysia

Tel: +60-3-89467475

Fax: +60-3-89467510

E-mail: clho@upm.edu.my

Supplementary Table S1. Two-way ANOVA analysis on the effects of species, treatment, and their interactions on the sulfate content of seaweed and agar. df, degrees of freedom; SS, sum of squares; MS, mean square; F, ratio of variance. **, highly significant (p<0.01).

Sources of variation	df	SS	MS	*F* value	*p* value
Sulfate content of seaweed					
Species	1	3563.1803	3563.1803	142.08	<0.0001**
Treatment	1	345.7054	345.7054	13.78	0.0021**
Species x treatment	1	62.8932	62.8932	2.51	0.1341
Sulfate content of agar					
Species	1	101.1477	101.1477	24.97	0.0001**
Treatment	1	48.2534	48.2534	11.91	0.0033**
Species x treatment	1	7.5345	7.5345	1.86	0.1915

Supplementary Table S2. Overview of the statistics of transcriptome sequencing.

Sample ID	Library size (bp)	Total no. of bases	GC content %	No. of raw reads	No. of clean reads	Percentage of clean reads, %
**CC**	193±31.90	4.67×10^9	52	5.18×10^7	5.14×10^7	99.56
**CT**	205±29.24	4.83×10^9	52	5.37×10^7	5.32×10^7	99.53
**SC**	193±32.95	3.35×10^9	52	3.72×10^7	3.70×10^7	99.74
**ST**	194±30.41	3.23×10^9	53	3.59×10^7	3.57×10^7	99.83

Supplementary Table S3. Reciprocal BLASTn search of *G. changii* and *G. salicornia* unigenes. The results are divided into homologous matches between the two species and genes that are only expressed in *G. changii* or *G. salicornia*.

**** in a separate excel file***

Supplementary Table S4. DEGs of *G. changii* and *G. salicornia* treated under sulfate deprivation in comparison to their respective untreated samples.

**** in a separate excel file***

Supplementary Table S5. Primer sequences of genes analyzed by qRT-PCR analysis.

Gene ID	Primer sequence (5'-3')		Primers Conc., nM	PCR product size, bp	PCR efficiency, %	R^2
	Forward	Reverse				
DV962495	ATTCGTGATGCCGTGACATA	TGCCAAACAAGTCCTTCACA	400	228	93.6	0.997
NODE4918	GGCAACCACAGGAAAGGAACC	CATTTTTCACCTCGGGCCATC	400	245	100.5	0.991
NODE1703	GGCAACAAAGCGTGTGAGACC	GGTGCGATATTGCATGG	400	330	98.5	0.990
GC_35	GAGACGTGGCGATTCTTTACG	GAAGTTAGCTCAACGCGCAAC	300	198	95.1	0.996
GC_2611	TGGTGGAGAAGGGCATTGTGA	TCTAAACACACTGGCGTCCGAC	300	196	100.6	0.994
GC_4791	AAGATCCTGCGGTGCGAAT	AGCGTCTGGAGGACAGCAAA	300	166	99.3	0.994
GC_4377	ATTCGACGCATCTGCTACGC	TACTGATGGGCGCTAACACG	300	159	100.1	0.996
GC_2437	TCTGGTTGCTCTTCGCATTG	CGAGGAAGAGGAGGGACGTT	300	187	103.8	0.993
GC_31	CCGAGTGGAATACCGGTGAA	CTGCCATCCACATTTCGTCA	200	181	96.0	0.997
GC_3015	ACGTCGTGGATCAGCTCGTT	TTTCGTTCGCCTTTTCCTCA	300	194	101.8	0.996
GC_774	CAGATTGTGTGCCGTTTGCT	GCACCACCTGATGCTCACCT	200	193	98.3	0.998
GC_45	CTGAAGGTCCGAGCGGTCTA	TCGGCTTCGAGCTTGATGAT	300	203	103.3	0.994
GC_4229	GTGATTCGTGCTGGCAGGTC	AAAGTGCGCGACTTGAGTTT	300	109	102.0	0.994
GC_13944	CGGAACGAACTTTGCTCCAT	CACGGAGCCACTTGTGTACG	400	199	97.9	0.993
GC_7491	TGTCGTGAAGAACGGATTGG	GCGTCACTTCTTTCTGCTATGG	300	254	95.6	0.990
GC_2026	AGCGTGGAATTCGATGGAC	CCAACACCTTTTGAGGCTGA	300	152	98.1	0.997
GC_3116	TGGACGTTATGGGCTTCAAC	GGCTCCAAGGAAAAGACCTG	300	181	96.2	0.997
GC_2731	TGCCGCACTCTGCACTAGAA	ACAGAACGACTAAGCGTCAGCA	300	136	98.6	0.989
GS_3854	GAATTGAACGCGGTGACAAC	GATCCAGCCTGCCGAAAAT	300	187	97.0	0.991
GS_4017	CGCCAGTTTGGATTCATCG	CACAGCAGCGTTGTCTCATG	300	181	93.0	0.992
GS_3763	TGAGTCGCAAAGAGATCTGACC	GTATGGGGTGCTCAACAACTCC	300	186	100.5	0.992
GS_8637	CGATGAGGAGAAGGCAAAGG	CAATTGCATCGACGTGAGGA	400	173	102.2	0.994
GS_6369	TCCGTTCTTGGAGAGGATGTCT	CACCCTCCTACCATCGTACCTC	300	198	102.3	0.990
GS_4697	CGTGTTGCAGCGTATGACAA	ACGAGCTCATCACGCACACT	200	210	97.6	0.994
GS_6497	CGTGGTTTGTTTCGGACAGA	TTCAACAGCATGGCGTAACC	300	190	92.1	0.990
GS_2523	CCAATGGAGAGGCACAGCTT	GTGCATACGCCATCCAAAAT	200	151	99.5	0.989
GS_6887	TGAGAAGGTGGCCATTCCTT	TTATGCCTTCGCACTTTCCA	300	130	100.8	0.998
GS_6282	GAAAAGCGTCATGCGACAGA	TAGGCCATGGCTTGTGATTG	200	214	91.8	0.996
GS_8540	CATTGGTAGCGGGAGCAAA	TTGCGAACTCTCCTGTCAGC	300	147	100.5	0.997
GS_5546	GATTTCCATTGCAGACCTTGC	GGAGCAGCGAACAGCACTAA	300	217	94.1	0.994
GS_4274	GTGAGAAGCGAAAGGGATGG	AAGTGTCCCTTGTCGTCGTG	300	174	102.1	0.983
GS_5748	AAGCGAGCTGTGAGCATACG	TGCTACCTCCCGCAAACTCT	300	225	102.2	0.994
GS_9077	TTGGAGAGCAGGTTGCGATC	CAACTTGCGAGCTGAAACGA	300	174	100.5	0.998
GS_1965	TGCAAGAGGCTATTGACCAGAA	GAAACGCTCACCGTCTTGCT	300	180	95.0	0.993
GS_7955	CCCTATGGCGACTGAAAAGC	TCTTCCCGACTTCACCCTGT	200	174	97.3	0.989

Supplementary Fig. S1. Cumulative number of unigenes in *G. changii* (a) and G. *salicornia* (b) with their top hit (generated by BLASTx against NR database) matches to sequences from red algae/other species at different cut-off RPKM values. The red algae here include *Chondrus crispus*, *Galdieria sulphuraria*, *Cyanidioschyzon merolae*, *Gracilaria gracilis*, *Gracilaria changii*, *Gracilaria tenuistipitata*, *Gracilaria salicornia*, *Gracilaria chouae*, *Gracilaria vermiculophylla*, *Griffithsia japonica* and *Gracilariopsis andersonii*. Collen *et al.* [1] revealed that the ratio of protein coding genes from *Chondrus crispus* that share sequence similarity to red algal (*Cy. merolae*, *P. yezoensis*, *Porphyridium cruentum* and *Calliarthron tuberculosum*) sequences to those that match to other organisms is 1.31:1. Since the sequence similarity search in this study was performed against NCBI NR database which contains a higher number of red algal sequences, a more conservative ratio of 2:1 was estimated, which is with a RPKM cut off at 15 for both *G. changii* (2.21:1) and *G. salicornia* (1.97:1).

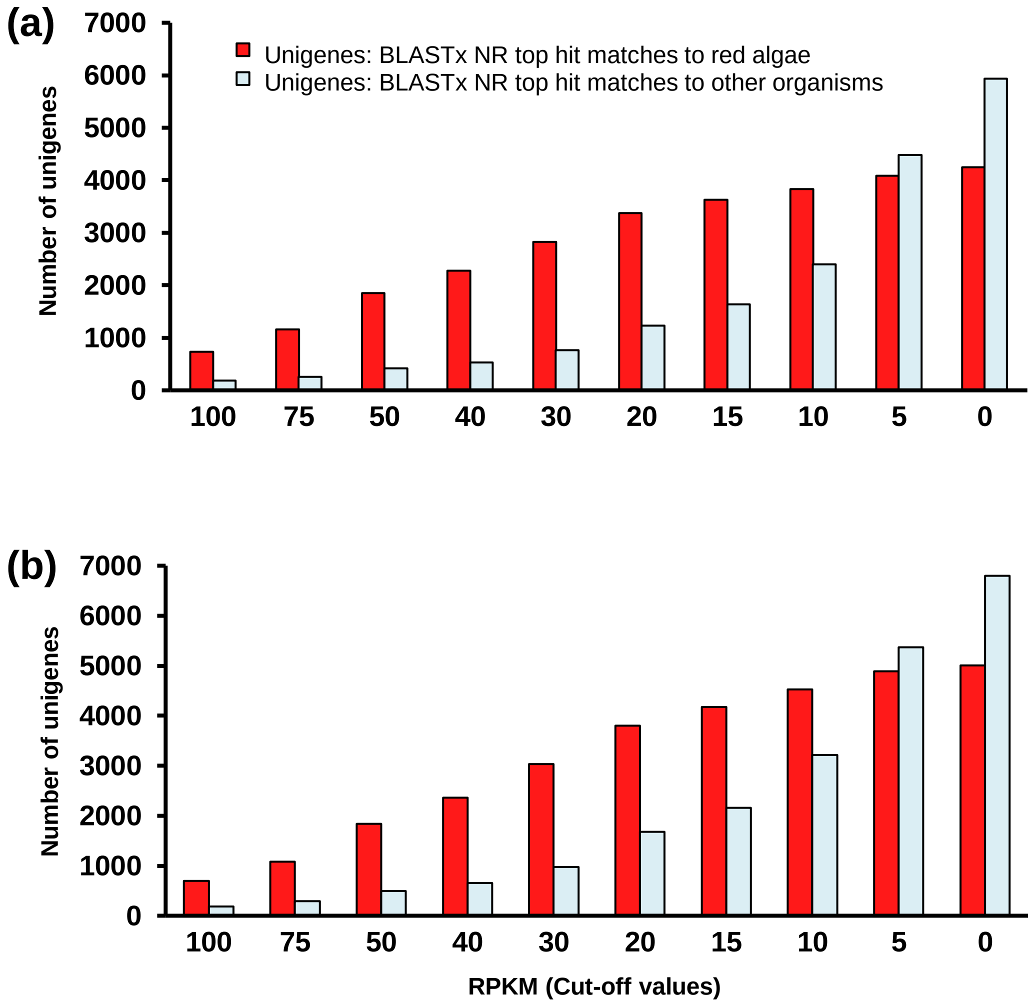

Reference:

1. Collen, J. *et al*. Genome structure and metabolic features in the red seaweed *Chondrus crispus* shed light on evolution of the Archaeplastida. *Proc. Natl. Acad. Sci. U.S.A.* 110, 5247-5252 (2013).
